# Grape Pomace for Feed Enrichment to Improve the Quality of Animal-Based Foods

**DOI:** 10.3390/foods13223541

**Published:** 2024-11-06

**Authors:** Francesca Blasi, Valentina Trovarelli, Luciano Mangiapelo, Federica Ianni, Lina Cossignani

**Affiliations:** Department of Pharmaceutical Sciences, University of Perugia, 06126 Perugia, Italy; francesca.blasi@unipg.it (F.B.); valentina.trovarelli@dottorandi.unipg.it (V.T.); luciano.mangiapelo@dottorandi.unipg.it (L.M.); federica.ianni@unipg.it (F.I.)

**Keywords:** agri-food waste, phenols, circular economy, fortification, food quality, livestock

## Abstract

In this review, the potential role of grape pomace (GP) as a tool for improving feed has been critically summarized, considering the findings of the literature of the last five years (2020–2024). The main applications of GP to the nutrition of different animals and the impact on derived foods (meat, milk and dairy products, eggs, fish) are discussed along with the major advantages and limits. Emphasis was placed on the phenols and fatty acids of GP, which are considered phytochemicals with health-promoting effects. Phenolic compounds increase the antioxidant potential of animal-based foods even if their content and profile are strongly related to grape cultivar and geographical origin. Unsaturated fatty acids, including linoleic and oleic acids, contributed to extending the shelf life of new products. Few approaches exploited chemometrics tools. Generally, GP showed a promising role in feed fortification, even if, in most cases, GP was key only if used in a correct percentage within a balanced diet and for an adequate administration time. From a multidisciplinary perspective, future research endeavors should prioritize a larger sampling, a deep phenol fraction characterization, and an appropriate chemometric approach.

## 1. Introduction

The growth of the world population has led to the intensification of food production processes, increasing the amount of organic solid waste produced annually. Furthermore, solid agro-industrial waste generated by agro-processing industries causes disposal difficulties and economic loss, negatively impacting wildlife [1]. According to the Circular Economy Action Plan, food waste can be included in the circular economy and should be considered for different reasons along the entire food chain [2,3]. Some of these by-products are rich in bioactive compounds such as fatty acids (FAs) and polyphenols, which are attractive for pharmaceutical, cosmeceutical, and nutraceutical perspectives.

One of the major contributors to solid waste generation in the food industry is wine production, especially in Italy, France, Spain, and California [4]. Managing by-products of the wine industry is a monumental task across the globe: *Vitis vinifera* L. grape is one of the main fruit crops cultivated worldwide, with an annual production of more than 67 million tons [5]. A total of 80% of the total grapes harvested are applied in the wine-making process [6], during which up to 200 kg of solid waste is generated for 750 L of wine produced. Of this solid waste, 60% consists of a mixture of grape skins and seeds, grape stalks, wastewater, and wine lees. This waste is called grape pomace (GP) [7].

Nowadays, unconventional extraction techniques, characterized by low energy, low solvent, and sustainability [8,9,10], are used to isolate bioactive compounds from GP for nutraceutical and pharmaceutical aims. Interestingly, Sodhi et al. [11] have introduced a microbial-based valorization of GP to be used as nutraceuticals to promote circular economy. Recently, the market trend of GP in sustainable cosmetics was reviewed by Castro et al. [12]. Another possible use of GP to prevent its accumulation as waste is in the fortification of food for humans or animals since GP is an important source of nutritional compounds and bioactives, including fiber and polyphenols [13]. The enrichment with GP of animal feed can reduce feed costs and improve the quality of the final food, with better profitability for farmers and lower environmental impact [14]. However, besides the benefits, using food waste as animal feed has some drawbacks, including a lack of safety and an unpredictable and highly variable nutrient profile [15].

This review describes the use of GP to fortify the diet of different animals (ruminants and non-ruminants) and evaluates the influence of this addition on the composition, quality, and shelf life of the resulting foods, considering the findings of the literature of the last five years (2020–2024).

## 2. Animal-Source Food and Impact on Human Health

European countries are key producers and exporters of livestock products, such as pig meat, poultry, milk, eggs, and fish. These products are widely consumed globally, although recently, the diet has been increasingly shifting toward plant-based nutrition, as reported by the Agricultural and Rural Development Department of the European Commission [16].

Consumption surveys across Europe show that meat, fish, eggs, and dairy have reached a plateau or are declining, except for poultry meat; pork remains the main meat consumed in Europe, as reported by Prache et al. [17]. Data from researchers in the animal production sector demonstrate that the quality of these products is directly related to animal feeding practices.

It has been observed that the human Western diet is deficient in *n*-3 polyunsaturated fatty acids (PUFAs) and their precursor, the α-linolenic acid (C18:3 *n*-3). Some fatty acids (linoleic C18:2 *n*-6 and α-linolenic acids) are essential, as the human body cannot synthesize them. This nutritional inadequacy is an aggravating factor in many chronic diseases and can lead to severe obesity with significant oxidative stress [18]. Excessive ingestion of some nutrients, such as atherogenic saturated fatty acids (SFAs), can cause adverse health effects [19].

The nutritional attributes of animal-source foods are evaluated based on their chemical composition and ability to cover human nutrition needs. One strategy to modify the composition of foods of animal origin to increase their health properties or shelf life is to fortify animal feed. The link between the ingested FAs and their profiles in animal body tissues and products is tight for monogastric and fish, while it is less close for ruminants due to the FA conversion processes within the animal body. Regarding mammalians, FAs secreted in milk are also modulated by de novo FA synthesis in the mammary gland [20].

The upcycling of by-products for animal feed is one of the strategies to reduce food–feed competition and dependency on imported feed resources. GP has attracted increasing interest within the food field due to its valuable composition, including nutrients and bioactive compounds (FAs, polyphenols, organic acids, vitamins, etc.) [21].

Polyphenols are a group of phytochemicals classified as flavonoids (e.g.*,* flavonols, isoflavones, and anthocyanins) and non-flavonoids (e.g.*,* phenolic acids, stilbenes, and tannins) [22]. It is known that an appropriate diet and lifestyle are essential to maintain well-being and prevent disease and that polyphenols, with their antioxidant properties, show potential beneficial effects on health [23].

In a recent review [24], some of the beneficial properties of GP are reported, such as anticancer, antidiabetic, hypolipidemic, and cardioprotective effects. The valorization of GP represents a challenge to solving waste management problems in the wine industry [25,26]. Another important reason to use GP is the technological aspect, as its phenols can react with superoxide anions, hydroxyl radicals, and lipid peroxide radicals, responsible for the rancidity of foods, thus acting as antioxidants and helping to prolong the shelf life of foods **[27]**. Based on these considerations, the use of GP as a food additive to develop new products, such as animal feed, has recently been proposed.

## 3. Grape Pomace: Overview of Chemical Composition

As reviewed by Antonić et al. [21], the proximate composition of grape pomace is highly variable, and it depends on the climate [26] and consequently on the geolocation [28], but also the grape variety [29] and wine-making procedures [30,31].

Antonić et al. [21] reported that the fat ranged from 1.14 to 13.90 g/100 g. For the eight varieties from Virginia, the lipids ranged from 4.62% to 12.5%, with linoleic acid (C18:2 *n-*6) as the predominant fatty acid (59.0–70.9%); in fact, it was widely reported that the oily part of the GP is rich in unsaturated fatty acids, including oleic (15.5–21.8%) and α-linolenic (1.05–2.85%) acids, too [30]. GP is a fiber-rich by-product with high variability of composition: total dietary fiber (17.88–88.70 g/100 g); insoluble components (16.44–63.70 g/100 g); and soluble components (0.72–12.78 g/100 g) [21]. Among non-extractives, lignin (25.2–44.5%) was the major component in white and red GP [30]. Glucan (8.04–12.7%) was the major structural carbohydrate, followed by xylan and minor amounts of other structural carbohydrates [30]. The total soluble sugars were between 7.79 and 108 g/kg, being known that the sugar content in grapes is quite high. The sugars most represented in pomace are glucose and fructose. Antonić et al. [21] reported that the glucose ranged from 0.21 to 26.34 g/100 g and fructose from 0.38 to 8.91 g/100 g. The protein content (3.57–14.17 g/100 g) is also variable. GP contains a considerable amount of amino acids (AAs), including tryptophan, 5-hydroxytryptophan, and L-dopa [9]. Regarding ash, Antonić et al. [21] reported a range from 1.73 to 9.10 g/100 g, and Jin et al. [30] from 4.32 to 6.60%. Cid et al. [32] showed that the most represented minerals were K, Fe, Zn, P, and Cu in seeds and skins of distilled and undistilled GP samples.

Among bioactive compounds, important components are polyphenols [33]. GP had a high content of phenolics because of the incomplete extraction during the wine processing [34]. TPC was generally quantified using the spectrophotometric Folin–Ciocalteu assay, showing a wide range dependent on several factors (i.e., grape variety, type of extract, etc.) [21,35]. For example, Mohamed Ahmed et al. [31] studied the chemical composition of GP (with seed and skin) from 10 varieties. They reported a TPC ranging from 75.41 to 147.51 mg GAE/100 g and identified ten phenolic compounds, among which protocatechin and chlorogenic were the most abundant. Recently, Mangiapelo et al. [36] have optimized a simple analytical workflow to characterize the phenolic fraction from GP. Among the identified compounds were gallic, protocatechuic, vanillic, syringic, and caffeic acids, along with catechin, epicatechin, resveratrol, quercetin, and kaempferol. Based on the above, it can be concluded that the chemical composition, including the determination of TPC, total antioxidant activity, and phenol profile, of GP used for the functionalization of feed must be included in research of this type, for which further efforts are needed to use this by-product for the production of value-added products, supplements or nutraceutical [36].

## 4. Grape Pomace As a Functional Ingredient in Animal Diet

In the following paragraphs, different animals fed a diet fortified with GP and the impact on the nutritional composition of derived products will be critically discussed, highlighting the strengths and weaknesses of the experimental approaches and the following analytical steps. Firstly, ruminants, including steers, lambs, cattle, and goats, will be reported, then non-ruminants, including poultry, pigs, rabbits, and fish.

### 4.1. Ruminants (Meat and Dairy)

**Figure 1:**
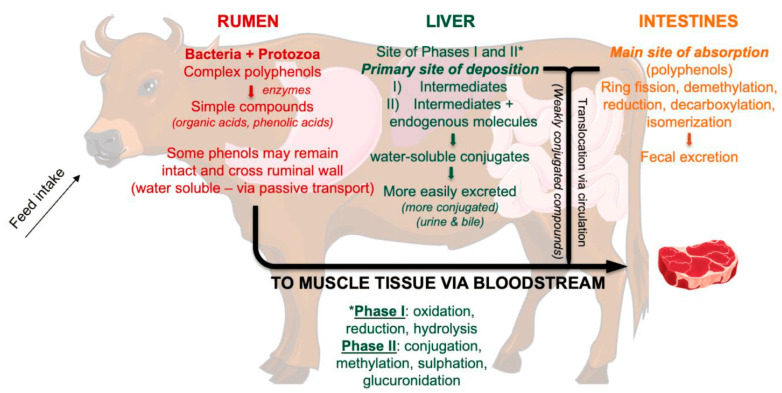
Phenolic compounds metabolism in ruminants [40].

In addition to meat, many consumers use dairy products derived from ruminants on a daily basis. The impact of feed added with different percentages of GP is variable for what concerns the meat quality, including marbling, color, and appearance, key determinants of consumer acceptability [37]. Table 1 shows research papers (2020–2024) focused on GP integration in the diet of ruminants. Among ruminants, in the period investigated in this review, only three research papers concerned meat from STEERS, in detail Jersey × Holstein [38] or Angus [39,40] breed, fed with diets containing GP. The bioavailability of polyphenols deriving from the small intestine is low since they undergo biotransformation in the body as if they were xenobiotics. Some phenols are absorbed in the rumen. Interestingly, some phenols may also come from the catabolism of AAs in ruminants (Figure 1) [40].

**Figure 2:**
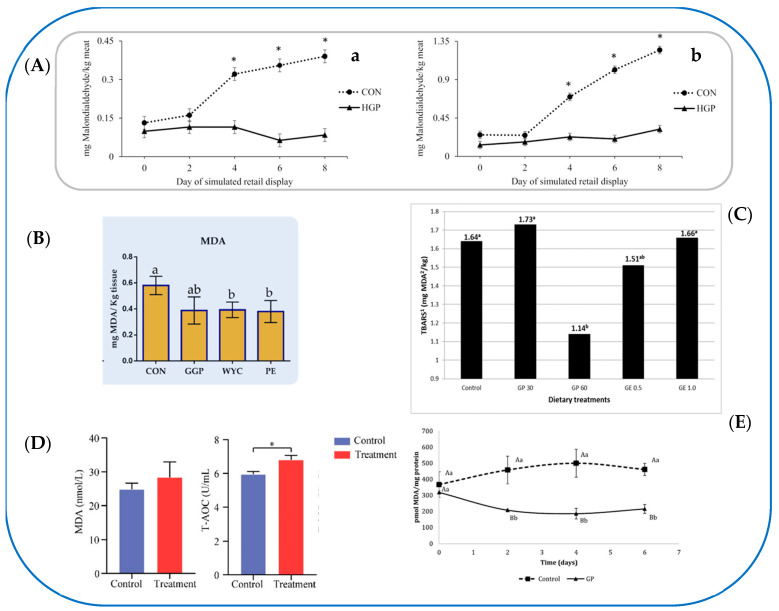
(**A**) Lipid oxidation over 8 d of simulated retail display for striploin ((**a**): *longissimus lumborum*) and top round ((**b**): *semimembranosus*) steaks from cattle fed a typical co-product-based finishing diet (CON) compared to a diet containing 58% (DM basis) grape pomace (HGP). Pooled SEM = 0.0257. Diet, *p* < 0.01; day of simulated retail display, *p* < 0.01; diet day of simulated retail display, *p* < 0.01. Error bars represent the standard deviation. * Significant difference between CON and HGP at *p* < 0.05 [38]; (**B**) lipid peroxidation index of breast muscle of broilers fed the four diets (control; CON, ground grape pomace; GGP, dried wine lees extract; WYC, and grape stem extract included in soluble starch; PE) at 42 days. Bars with different superscripts (a, b) between dietary treatments differ significantly (*p* < 0.05) according to the analysis of variance (ANOVA) using post hoc multiple range test when appropriate [57]; (**C**) yolk lipid oxidation (SEM 3 = 0.149, *) measured in four-month-stored eggs of laying hens fed diets containing grape pomace (GP) or grape extract (GE) at different concentrations. Different letters (a, b) indicate significant differences (*p* < 0.05). 1 TBARSs: thiobarbituric acid reactive substances. 2 MDA: malondialdehyde. 3 SEM, standard error of means; each value represents the mean of nine samples per dietary treatment (three samples per replicate); each sample resulted from the pool of two yolks. * *p* < 0.05 [63]; (**D**) serum levels of indicators including MDA and T-AOC were measured by using ELISA kits (*n* = 6) [68]; (**E**) muscle thiobarbituric acid reactive substance (TBARS) content in fillets of *D. labrax* fed on experimental diets (C: control; GP: 0.4% grape pomace) over cold storage (4 °C) at times: 0, 2, 4, and 6 days. Values are presented as mean ± SD. Comparisons of values between time within each diet are noted with small letters; comparisons of the same sampling points among different diets are noted with capital letters. Values not sharing a common letter differ significantly with *p* < 0.05. Reproduced from Ref. [83], which is an open-access article distributed under the Creative Commons Attribution License (CC BY 4.0), Springer, https://creativecommons.org/licenses/by/4.0, accessed on 5 October 2024.

Interestingly, Arend et al. [38] fed crossbred steers a finishing diet (by-product-based typically fed in the Pacific Northwest: GP 7.54%, apple pomace 7.58%, bakery waste 9.02%, whey 5.69%) as control (CON) and a diet containing 58% of GP (HGP). The FA profile, lipid oxidation, and meat quality were evaluated. The use of HGP silage led to a decrease in carcass weight with a reduction in the yield due to an energy deficiency, caused by the lowering of starch content and the increase in fiber content in the diet. To demonstrate the positive impact of the GP bioactives on health, the high content of phenolic compounds in the HGP diet led to a decrease in lipid oxidation (malondialdehyde—MDA—value) measured at the muscle level (steaks from *Longissimus lumborum* and *semimembranosus* muscle) (Figure 2A(a,b)).

Moreover, the PUFA content in HGP meat increased compared to CON meat (10.3 vs. 5.87%, respectively), and in particular the content of linoleic acid, rumenic acid (C 18:2 *c*9*t*11), and total conjugated linoleic acids (CLAs, ∑18:2 *c*9*t*11, 18:2 *t*9*t*12, and 18:2 *t*10*c*12). The authors hypothesized that this result was due to the higher content of polyphenols introduced with the HGP diet. They act by limiting the biohydrogenation pathways of linoleic acid in the rumen. The research of Arend et al. [38] showed that feeding cattle with a high amount of GP could limit growth performance, even if an enhancement of the sensory quality and FA profile of meat increased consumer acceptability.

Another group of researchers [39] compared the effects of two different diets (one added with dried citrus pulp—DCP—and one with dried GP—DGP—each at 150 g/kg) on the meat from Angus steers, evaluating the FA and sensory profile of intramuscular fat. Regarding the lipid profiles, the percentage of oleic acid was greater in beef fed control compared to the diet supplemented with citrus and GP. Conversely, the percentages of linoleic, arachidonic, α-linolenic acids, total CLAs, *n-*3 PUFAs, and total PUFAs in GP-fed beef were higher than in the control. Interestingly, the authors analyzed by solid-phase microextraction (SPME) and gas chromatography–mass spectrometry (GC/MS) the profile of volatile compounds in beef, identifying 44 compounds, which differed according to the dietary treatments. However, the authors found that none of the hydrocarbons and organic acids identified were influenced by diet. GP-fed beef had greater concentrations of heterocyclic and sulfur compounds than the control group. Except for tenderness, other attributes (i.e.*,* aroma, flavor, and texture) were similar across the diets. The control beef was 10% more tender than GP-fed beef, but the diet did not affect the major sensory attributes [39].

The meat quality of Angus cattle fed three different diets was also studied by Krusinski et al. [40]. During the 2-year study, cattle were fed a diet consisting of pasture grass (GRASS), another containing mixed cereals (GRAIN), and a third containing mixed cereals and 5% GP (GRAPE). In addition to the FA profile, the authors also evaluated the amount of minerals, vitamin E, and other metabolites in the beef. Overall, meat from GRASS-fed cattle showed the best nutritional profile with a lower *n*-6/*n*-3 ratio and higher levels of long-chain *n*-3 PUFAs, vitamin E, zinc, iron, and secondary metabolites (including citric, succinic, and fumaric acids), compared to meat from cattle fed other diets. Interestingly, the meat of cattle fed with GRAPE was halfway between the cattle fed with GRASS and GRAIN for the concentration of total *n*-6 PUFAs, eicosapentaenoic (EPA, 20:5 *n*-3), and DHA. Some weak points of this study were highlighted by the same authors. In fact, they stated that probably the percentage of GP (5%) was too low to appreciate a substantial improvement in the nutritional profile of the meat, as well as the small final sample size (*n* = 4) for GRAPE, leading to high standard error values [40]. In appearance, the paper of Krusinski et al. Reference [40] seems the best among the above cited for the high number of animals and period of feeding (2 years), even if it can be observed that the sample collection shows some limits/lacks, among which are different numbers of samples among GRASS, GRAIN, and GRAPE in the two years; different age of animals; and no GRASS samples were left for phytochemical analysis.

Taking into consideration the impact of the chemical composition of the animal diet and the presence of phenolic compounds in GP, in the three studies mentioned above [38,39,40], the nutritional compositions of the diets were also reported, even if only Arend et al. [38] and Tayengwa et al. [39] showed the proximate composition, the FA profile together with TPC, free phenols, tannins, and non-tannins of the diets. Krusinski et al. [40] showed only a simplified diet profile showing crude protein, neutral detergent fiber, acid detergent fiber, energy, and FA profile. They did not evaluate TPC or similar parameters, but a simple phytochemical profile showed the presence of caffeic, *p*-coumaric, *p*-hydroxybenzoic, gallic, and vanillic acids. None of the three cited papers reported details on GP, such as the grape variety, the duration of the fermentation process, or whether the by-product was submitted to an ensilage process (conditions, time, …). In our opinion, these aspects are important for a more accurate evaluation of the final goal because the TPC, the antioxidant activity, and the phenol profile of the GP also depend on these aspects, which then influence the quality of the meat.

Among ruminants, lambs must also be cited. As reported in previous studies on steers, the lipid fraction of lambs fed with GP was also analyzed by other authors [42,43,44] as a quality parameter of meat.

Maciel et al. [43] studied the effect of different concentrations of GP (150, 300, and 500 g/kg DM) in the feed of young lambs compared to a control group. The study showed that increasing the percentage of GP added to the diet decreased the daily weight gain of the animals. Moreover, no significant differences were detected in the composition of the meat except for the FA profile; in fact, it was highlighted that intramuscular linoleic acid increased (from 3.48 to 6.33 g/100 g fat) as the level of GP in the diet increased. Furthermore, an increase in *n*-6 PUFAs and a reduction in palmitic acid (C16:0) was also observed [43], positive factors for human health, except for the increase in the *n*-6/*n*-3 ratio.

Unlike the previous paper, Islam et al. [51] observed the effects of supplementing sweet sorghum with grape seeds and not with GP, which generally contain seeds, although this is not always specified by the authors. They evaluated the carcass parameters, meat quality, AA, and FA composition of young lambs divided into four groups, each fed a different diet. One group served as a control and received sweet sorghum silage, another sweet sorghum hay, and the last two groups received sweet sorghum silage with grape seeds and sweet sorghum hay with grape seeds (each 10 g/kg DM). Among the parameters analyzed, the color of the meat showed an increase in redness and a decrease in methionine concentration in lamb fed sweet sorghum with grape seeds. Lambs consuming sweet sorghum silage showed increased water content in their meat and a lower concentration of *n*-6 PUFA and *n*-6/*n*-3 PUFA ratios compared to lambs consuming sweet sorghum hay. It is interesting to note that SFA content, as well as the thrombogenic index (TI) in meat, were the lowest in lambs consuming sweet sorghum silage with grape seeds added, while the contents of EPA and DHA tended to increase [44]. For the first time, two indexes generally used to verify the quality of the fat in animal-source foods, i.e., TI and the atherogenic index (AI), were calculated, together with some FA ratios (*n*-6/*n*-3 and PUFAs/SFAs).

These two indexes (TI and AI) were also evaluated by a research team with wide expertise in feed integration [42]. They studied the effects of GP supplementation (10%) in lamb diets, reporting the chemical composition together with FA profiles, TPC, and antioxidant activity. The GP used for the experimental diet showed a TPC of 73.36 mg GAE/g and an antioxidant activity of 496.12 µmol TE/g. The 10%GP diet showed values of TPC and antioxidant activity significantly higher than the control (2.44 mg vs. 2.16 mg GAE/g; 52.41 µmol TE/g vs. 45.09 µmol TE/g). They evaluated some meat quality parameters (meat color, drip loss, cooking loss) and the chemical composition of meat (FA, volatile profiles, and lipid oxidation). GP supplementation was effective in improving the FA composition; for example, a significant rise in the levels of CLAs (1.22% vs. 1.07%) was observed together with the oxidative stability of lamb meat [42]. Interestingly, this article is one of the few papers that evaluate meat cooking (water bath until an internal temperature of about 75 °C is reached) and subsequent storage at 4 °C. These authors characterized by SPME-GC/MS the volatile profiles of cooked meat samples, identifying 16 compounds belonging to the categories of aldehydes, alcohols, ketones, and phenolic compounds. The results showed that hexanal was the most represented compound, and significant differences were found only after 5 days of storage (a decrease in 1-pentanol and 1-octanol and an increase in 2-octen-3-ol and 2-octen-1-ol). Most of these molecules, impacting the aroma and taste of the product, are prone to accumulation during heat treatments. Volatile compounds are mainly released by Maillard reactions, lipid oxidation, or interactions between Maillard reaction products and oxidation products [42]. This study, as well as that of Tayengwa et al. [39], showed that changes in the diets of animals induced variations in the accumulation of volatile compounds in meat. It follows that this kind of analysis should also be included in other studies on the same topic, and instead, generally, it is not carried out either on the product as is or on the cooked product.

Unlike the three previous studies on lambs [42,43,44], the research of Flores et al. [41] and Vieira et al. [45] did not focus on meat lipids and hypothetical lipid markers. Flores et al. [41], for example, aimed to evaluate productive performance, meat quality, and blood and liver parameters (i.e.*,* aminotransferase aspartate, alanine aminotransferase, γ-glutamyltransferase, hematocrit) of lambs fed diets consisting of increasing levels of GP silage (from 0 to 50% forage/concentrate ratio) as a partial replacement of maize silage. They also evaluated the chemical composition of the experimental diets, showing that TPC increased as the concentration of GP increased (from 8.95 to 15.06 mg GAE/g at 0% and 50% GP, respectively), as well as the content of tannins (from 0.69 to 1.52 mg/g) and anthocyanins (from 0 to 93.50 mg malavidin-3-glycoside/g). This study did not reveal any significant variations in the quality parameters of the meat: marbling, pH, and color. Only a linear reduction in weight was detected as the GP content in the diets increased, probably due to the increased fiber content, which may decrease the feed passage rate through the digestive system of animals, increasing feed retention time and reducing voluntary feed intake. In this regard, the authors determined fiber composition and found that the amount of the acid detergent lignin increased from 5.22 to 18.81%. Despite this, one of the weak points is that the chemical composition of the meat was not evaluated; in fact, they reported TPC of blood and liver (0.23–0.44 mg GAE/g), but the phenol content/composition or the FA profile of meat were not evaluated [41].

Vieira et al. [45] studied the effect of supplementing the diet of lactating sheep with GP compared to vitamin E, a phytochemical known for its ability to prevent lipid oxidation [52]. Sheep received different diets, one added with vitamin E (500 mg/kg) and two with GP (5% or 10%). Interestingly, they evaluated the shelf life of meat during storage in retail sale conditions (packaged in a modified atmosphere with high oxygen content) by analyzing the microbiological, physical–chemical, and sensorial characteristics of the product. Despite the lack of a clear evolution of colorimetric coordinates, the metmyoglobin % was influenced by the presence of vitamin E and GP. Indeed, after 10 days, the lambs from sheep fed GP and vitamin E showed a lower percentage of metmyoglobin, a lower lipid oxidation (evaluated by TBARS, thiobarbituric acid reactive substances), and a better score on sensory perception than control. The authors concluded that GP was as effective as vitamin E in preventing spoilage during retail storage and that supplementation with 5% GP was more effective than supplementation with 10% GP in preserving meat quality [45]

In addition to meat, ruminants are the major producers of milk and dairy products [51]. Therefore, it is also interesting to investigate the nutritional impact of GP-enriched feed on these products. Firstly, it should be noted that no research relating to milk and dairy products from steers fed with GP supplements was published in the period covered by this review (2020–2024), but only a few reviews [53,54]. Among interesting remarks, Correddu et al. [53] reported that GP exerted positive effects on the FA profile, resulting in an improvement in the nutritional composition of cow milk fat, with a reduction in SFAs and an increase in MUFAs and PUFAs.

**Figure 3:**
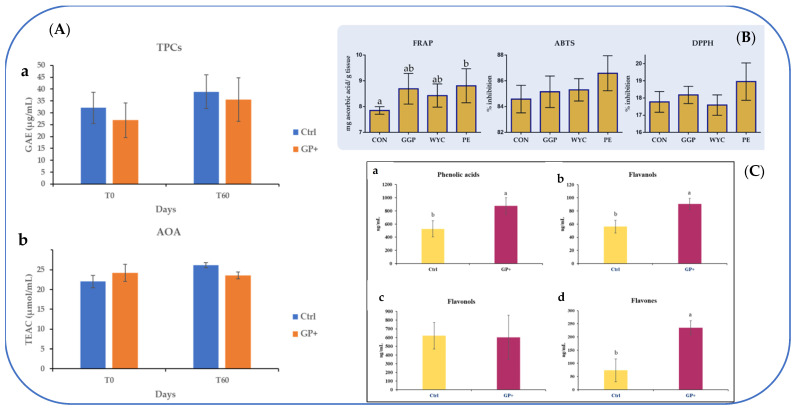
(**A**) Total Phenolic Compounds (TPCs) (**a**) and Antioxidant Activity (AOA) (**b**) at the beginning (T0) and after 60 (T60) days of trial in raw milk samples obtained from ewes fed a standard diet (Ctrl) and grape pomace diet (GP+). GAE = gallic acid equivalent; TEAC = trolox equivalent antioxidant capacity. Data are reported as mean ± SE for *n* = 23. Differences between Ctrl and GP+ were not significant (*p* > 0.05) [46]; (**B**) total antioxidant capacity index of breast muscle of broilers fed the four diets (control; CON, ground grape pomace; GGP, dried wine lees extract; WYC, and grape stem extract included in soluble starch; PE) at 42 days. Bars with different superscripts (a, b) between dietary treatments differ significantly (*p* < 0.05) according to the analysis of variance (ANOVA) using post hoc multiple range test when appropriate [57]; (**C**) main classes of phenolic compounds: (**a**) phenolic acids, (**b**) flavanols, (**c**) flavonols, and (**d**) flavones, identified in raw milk samples obtained from the control group (Ctrl) and experimental group (GP+). ^a,b^ Means with different uppercase superscript letters are significantly different (*p* < 0.05). All data are reported as least square means ± SEM (*n* = 10 for each group) [48].

Many papers regarding the supplementation of feed with GP were published by the research group of Bennato et al. [46,47,48]. In the first study, the composition of milk of sheep fed with 10% GP was analyzed [46]. The chemical composition of feed and milk and the TPC and antioxidant capacity (Figure 3A) were evaluated, even if no significant differences were found between the GP-enriched diet and the control diet. Regarding the chemical composition of the milk, it is interesting to note a lower lactose content in the 10% GP group compared to the control.

The inclusion of GP in the diet modified the milk FA profile, and 60 days later, significantly higher values of MUFAs and lower levels of medium-chain saturated fatty acids (MCSFAs) were found. Furthermore, the results showed that SFAs, PUFAs, short-chain saturated fatty acids (SCSFAs), and long-chain saturated fatty acids (LCSFAs) were not modified. A modification of the desaturation index of C14 (decreasing) and C18 (increasing) was also detected. Moreover, differently from all the above-cited papers, in this study, particular attention was paid to the determination of the total caseins (i.e.*,* α-casein, β-casein, k-casein) and whey proteins (i.e.*,* β-lactoglobulin and α-lactalbumin) analyzed by electrophoresis. The authors reported that in milk obtained from sheep that have integrated GP, no significant differences in casein and whey protein contents were found between the start of the experiment and the end (60 days later) [46]. Using the same animals (i.e., number of animals, feeding, time) reported in the paper of Bennato et al. [46], the authors published another work based on the characterization of the phenolic profile in ewe milk and its reflection on antioxidant and anti-inflammatory status [48]. It was the first paper reporting details on these aspects. They carried out an in-depth analysis by liquid chromatography–electrospray ionization–tandem mass spectrometry of phenolic compounds both of diets and raw milk samples, detailing, respectively, 23 and 22 compounds belonging to phenolic acid, flavanol, flavonol, and flavone classes (Figure 3B). The authors observed that the 10% GP diet enriched the phenolic profile of milk with a higher level of phenolic acids (gallic, syringic, ellagic, protocatechuic, chlorogenic, and 4-hydroxybenzoic acids), flavonols (epicatechin), and flavones (naringenin and orientin), and a lower content in flavonols (quercetin, quercetin-hexoside, and myricetin) compared to the control. The authors, however, did not detect a significant impact on milk antioxidant and anti-inflammatory status assessed by enzyme-linked immunosorbent assay. Only a lower activity of collagenases type IV (MMP-9) was found in the 10% GP group [48].

The milk of the same ewes of the studies shown by Bennato et al. [46,48] was used to obtain whey and ricotta [47]. In this case, they evaluated the effect of GP supplementation on the lipid profile, TPC, and antioxidant capacity of whey before (WBR) and post (WPR) ricotta cheese-making, and ricotta produced at different ripening times, after 1 (T1) and 5 (T5) days of ripening at 4 °C. Regarding the FA profile, no significant variations of some lipid parameters, including MUFAs and PUFAs, were observed in WBR and ricotta T1. However, in whey samples, lower levels of odd-chain FAs, such as pentadecylic (C15:0) and margaric (C17:0) acids, stearic, myristoleic, and linolenic acids were also observed. Conversely, higher levels of myristic and vaccenic acids were found. In ricotta, lower levels of C15:0, C17:0, and C14:1 were reported. Higher levels of vaccenic acid were observed, on the contrary, in ricotta samples obtained with GP supplementing. Furthermore, lower desaturation indexes, DIC14:0 and DICLA, and higher DIC18:0 were observed in whey and ricotta added with GP. Moreover, GP did not alter the main whey proteins (lactoferrin, serum albumin, casein, β-lactoglobulin, and α-lactoalbumin). Conversely, significant variations were observed in color (lower lightness and a* parameter). The TPC of whey after ricotta production obtained from ewes fed with GP supplements was higher than the control (26.47 µg GAE/mL vs. 21.57 µg GAE/mL), as well as the total antioxidant capacity value (TAC; 10.49 µmol/mL vs. 9.03 µmol/mL). The same trend was confirmed for ricotta at T1 and T5 ripening times. The reduction in hexanal in ricotta during the ripening suggested better oxidative stability. The results obtained in this study suggested that the supplementation of GP in the ewe’s diet did not worsen the quality parameters of dairy products but rather improved their oxidative stability [47].

For the first time, Antunović et al. [50] evaluated the effect of grape seed cake supplementation (GSC5: 5% DM at 75 g/day; GSC10: 10% DM at 150 g/day) on lactating goats In particular, metabolic challenges, quality, and quantity of milk produced were evaluated. Both GSC5 and GSC10A showed higher milk production (no significant differences) and higher protein, lactose, and fat milk content. Instead, milk from the GSC10 group showed significantly higher superoxide dismutase (SOD) and glutathione reductase (GR) activity, as well as reduced glucose concentration and value of somatic cell count. The feeding did not lead to significant differences in hematological/biochemical indicators, except for the content of β-hydroxy-butyrate, a compound related to a higher energy value of GSC-containing feed. The chemical composition of the diets was also analyzed, and they found that TPC increased with the increase in GSC% (from 56.06 to 812.06 mg/kg GAE, control vs. GSC10), as well as the amount of anthocyanins (from 114.23 to 240 mg/kg, control vs. GSC10). The authors concluded that this supplementation, therefore, appears to decrease the oxidative stress caused by breastfeeding [50]. This research did not consider the lipid fraction, and the main weak point is that neither the diets nor the final products were evaluated considering the detailed phenolic profile.

Goat’s milk was also studied by Renna et al. [49]. They investigated the impact of dietary dried GP (150 g/day) on milk yield, composition, and FA profile of milk produced by goats, randomly assigned to the GP group or the control group. Milk fat content was lower in the first than in the control group (32.4 vs. 33.1 g/100 g), while protein and lactose contents were slightly higher. A detailed analysis of FAs was performed by a high-resolution gas-chromatography–flame ionization detector with 77 compounds identified. There was no significant impact on butyric, caproic, caprylic, and capric acids and also on the major trans isomers, including vaccenic (C18:1 *t*11), linoleic and α-linolenic acids, and long-chain *n-*6 and *n-*3 PUFAs. However, rumenic acid in milk fat was significantly reduced (0.300 vs. 0.359 g/100 g of total FAs). This study supports that up to 6% of GP can be safely included in conventional dairy goat diets without compromising the production or significantly altering the milk FA profile. It would have been interesting if these authors had also included the phenolic content of the two diets and the milk obtained from goats [49].

### 4.2. Non-Ruminants (Meat and Dairy)

#### 4.2.1. Poultry (Meat and Eggs)

About non-ruminant animals, this paragraph will provide an overview of GP integration in poultry, pig, and rabbit feed. Poultry includes avian species, namely chickens and ducks, from which meat and eggs can be obtained for human consumption. Table 2 shows the research papers (2020–2024) focused on GP integration of the non-ruminant diet.

Among the research papers of the reviewed period (2020–2024), the main ones are those on poultry breast meat. Jurčaga et al. [55] evaluated the effect of integrating red GP (1, 2, and 3%) on lipid oxidation of thigh and breast meat of broiler chicken (Ross-308). After 42 days, the study did not reveal any significant differences regarding the oxidative stability (measurement of malondialdehyde, MDA) of the GP group compared to the control, nor any negative effects [55]. Similarly, in the paper of Haščík et al. [56], the same samples used by Jurčaga et al. [55], i.e., thigh and breast meat of broiler chickens fed with 1, 2, and 3% GP fresh matter, were analyzed for their AA and FA content. The authors concluded that the AA profile of breast muscle was not significantly affected. In the thigh muscle, however, significant differences in the content of threonine, valine, methionine, cysteine, and histidine were observed, especially in males. FA profile results showed that GP influenced monounsaturated oleic acid in breast muscle, which had the highest content in all experimental groups compared with the control group.

The same type of broiler chicken (Ross-308) was also used in the experiments of Mavrommatis et al. [57], Bonos et al. [58], and Thema et al. [59]. Among these papers, the first was the only one that studied the composition of GP phenolic fraction and the antioxidant status (blood plasma and breast muscle) of broiler chickens fed diets supplemented with wine-making by-products [57]. They determined the TPC, TFC, total tannin, and antioxidant potential of ground GP (GGP: 25 g/kg), wine lees extract (WYC: 2 g/kg), and grape stem extract (PE: 1 g/kg). They characterized by ultra-high-performance liquid chromatography coupled to mass spectrophotometry (UHPLC-MS/MS), the phenolic composition of all grape by-products used in broilers’ diets and for GGP revealed the presence of procyanidin B1, epicatechin, quercetin, and quercetin-3-β-D-glucoside. They also studied the activities of antioxidant enzyme indicators of oxidative stress, such as catalase, glutathione peroxidase (GPX), SOD, and TAC at liver, muscle, and blood levels. The growth performance of the chickens, measured after 42 days, i.e., the weight gain following the diets consumed, was also determined, even if it was not considerably affected. The relative transcript level of GPX1 and SOD1 of broilers was prone to improve in the liver of the WYC-fed group, while NOX2 decreased in the PE-fed group. The TAC value was significantly higher in the breast muscle of PE-fed broilers (Figure 3C), while the MDA value was significantly lower in both WYC- and PE-fed broilers (Figure 2B). The study by Mavrommatis et al. [57] showed that PE and WYC were promising feed additives in broiler diets for improving the oxidative status of organisms and meat. The main weakness of the work was the lack of phenol characterization and FA profile of the final product, i.e., meat.

An interesting aspect not treated by the previous papers was the preliminary treatment for administering GP to the animals. Bonos et al. [58] evaluated the use of a novel silage created by combining three mass-produced agro-industrial wastes as feed. The two experimental groups of chicks were fed 5% and 10% silage based on agro-industrial waste products, including GP, olive mill wastewater, and deproteinized feta cheese whey. They evaluated meat color, oxidative stability, and FA profile. The analysis of color showed that the breast meat of the 10% silage-treated group had a higher yellowness compared to the 5% silage-treated group. The chemical composition and the oxidative stability (MDA and TBARS parameters) of thigh meat did not differ among the treated groups. A similar observation can be made for the TPC of breast and thigh meat. Only in the thigh meat of the 10% silage-treated group the TPC was higher than the control (4.02 g/L vs. 2.88 g/L). FA analysis of the breast meat identified some differences in the animal groups: myristoleic acid was absent in the 10% silage group, and palmitic acid was also lower in the 10% silage group compared to other groups. SFAs were higher in control compared to 10% silage (34% vs. 31%), while the trend was the opposite for UFA, which was higher in silage 10% than in control (68% vs. 66%). This showed that the increase in silage containing GP increased the concentration of UFA while the percentage of SFAs decreased [58].

Sometimes, GP was used in combination with other bioactive products. Thema et al. [59] recently used red GP and aloe vera gel to improve stocking density-induced stress in broiler chickens. They studied the combined effect of aloe vera gel administered through drinking water and red GP powder (30 g/kg) on growth performance, physiological traits, welfare indicators, and meat quality. Dietary treatments contained 30 g of GP/kg diet plus either 1, 2, 3, and 4% of aloe vera gel in drinking water. The color characteristics and the pH value were measured to evaluate the meat’s quality. Cooking loss, drip loss, and water-holding capacity were also determined. As a result, the simultaneous treatment of broilers with 30 g GP/kg of diet and 1% aloe vera gel ameliorated their growth parameters and body weight. The treatments also altered some health indicators, gait scores, and meat quality attributes. The performance and physiological characteristics of broilers were not improved with higher doses of aloe vera gel. The sore point of the study of Thema et al. [59] is that the chemical composition, FA and AA profiles, and phenol characterization of meat were not determined.

Another type of chicken (Ross-508) was used by Bennato et al. [60]. They evaluated the effect of GP dietary intake (2.5%, 5%, and 7%) on nutritional quality, lipid oxidation, and volatile profile of breast meat. No significant differences were found for pH, cooking loss, and meat brightness, while GP intake was positive for reducing drip loss, yellowing, and maintaining meat redness. Supplementation led to a decrease in meat lipid oxidation, a result also confirmed by the volatile aldehyde decreasing after 7 days of storage. Regarding the FA profile, a reduction in SFAs and a significant increase in linoleic acid concentration emerged in the samples from animals fed 5% and 7% GP [60].

Two papers reported studies on animals less investigated for this type of experiment, i.e., geese and quails [61,62]. In the work published by Drotárová et al. [61], the FA profile in the meat of fattening geese fed with GP supplementation was analyzed. At the end of the experiment, the average weight of the experimental group was greater than that of the control group, with an increase in the average daily weight of 5.2% and liver weight of 22.5%. Regarding the FA profile of meat, the content of MUFAs increased by 2.5% (oleic acid by 3.0%), while a reduction in PUFA (1.5%) and SFA (3.2%) contents was observed. However, regarding the content of PUFAs, MUFAs, and SFAs, no significant effect on the abdominal fat of geese was found. The paper of Drotárová et al. [61] is novel for the scope of application on geese, even if the authors did not investigate the GP phenolic composition and the antioxidant properties of the final product. Sur et al. [62] studied the effects of grape seed powder and meal (GSP and GSM, respectively) on various parameters, including blood biochemistry, carcass FAs, and antioxidant status, of Japanese quails reared under high stocking density. They found that neither GSP nor GSM (3%) reduced biological markers of oxidative stress [62].

Another food product derived from poultry and widely used worldwide is the EGG. Romero et al. [63] studied the effects of feeding laying hens diets added with 30 or 60 g/kg of GP and a control diet (added with 100 mg/kg of vitamin E). Moreover, a diet containing grape extract (GE, 0.5 or 1.0 g/kg) was also taken into consideration for the first time. The productive performance of the hens, the egg quality, and the chemical composition were evaluated, with particular attention to the TPC and the FA profile. Furthermore, the oxidative status of the lipid species was also determined. No significant differences were found between dietary treatments for egg production (83.8%, on average), although mean egg weight was lower (67.5 g vs. 68.5 g) for dietary treatments with GP or GE, compared to control hens. Regarding the quality parameters, all experimental treatments resulted in a higher yolk color score (8.12 vs. 7.341) compared to the values measured in eggs of control hens. Dietary inclusion of GP (30 and 60 g/kg) and GE (1.0 g/kg) increased the albumen Haugh units (80.8 vs. 76.4) compared to the control treatment. However, no significant effect was observed between the different dietary treatments for shell thickness. Regarding the FA profile of the yolk, the dietary inclusion of GP (both at 30 and 60 g/kg) reduced the amount of SFAs (31.9% vs. 32.9%) and MUFAs compared to the control group. On the contrary, the percentage of *n*-6 PUFAs increased (28.9% vs. 25.7%) compared to the ω-3 series. Lipid oxidation measured by a TBARS assay showed that eggs from GP-supplemented chickens, stored for 4 months, had a lower concentration of MDA than the control (Figure 2C). In the study of Romero et al. [63], total extractable polyphenols and proteins of the dietary treatments and the hen excrements were also evaluated to assess the digestibility of proteins and phenol and to know how many phenols the hens were able to assimilate at the intestinal level. The results of their investigation indicated that laying hens can utilize GP phenolic compounds in the gut. To the authors’ knowledge, this was the first research work in which the digestibility of polyphenols in laying hens was measured, and the shelf life of the eggs was evaluated.

The same type of hens as Romero et al. [63] were used by Tufarelli et al. [64]. They evaluated the effect of dietary flaxseed meal (FSM) supplemented with dried tomato and dried GP (DTP and DGP, respectively) on various parameters, among which performance and antioxidant status. Interestingly, they applied a little-used approach consisting of a randomized design to study the impact of different percentages of FSM, DTP, and DGP. The authors found that FSM and DTP supplements had no significant effect on most hen performance indicators and egg quality parameters, although significant improvements when feeding 15% DTP and 5% DGP on egg traits, especially egg yolk color that plays a key role in consumer’s choice, were observed.

Recently, Herranz et al. [66] administered 50 g/kg of GP in the standard feed of hens (ISA White and ISA Brown) to establish whether this supplement could enrich eggs with antioxidants, evaluating the internal and external egg quality parameters. The authors reported the chemical composition and TPC value (0.728 g GAE/kg of added diet vs. 0.644 of control) of the diets. In the group fed with GP, an improvement in some egg quality parameters, such as the albumen Haugh units and the yolk color score, was observed. However, thinner eggshells were also found, although these changes were not reflected in the mechanical parameters of the eggs. Interestingly, egg yolks from hens fed the GP diet had a higher gallic acid content than eggs from hens fed the control diet, thus suggesting the potential transfer of GP polyphenols to eggs. A higher amount of α-tocopherol in ISA brown egg yolks compared to eggs laid by white ISA hens was found, while an opposite trend was observed for γ-tocopherol. Differences between the strains were also highlighted for other parameters, indicating the importance of genetics on egg quality. Although the diet containing GP was richer in unsaturated fats and, therefore, more prone to lipid oxidation, it did not lead to reduced concentrations of α- and γ-tocopherol in egg yolks [66].

Another type of hen (Lohman Brown Lite) was used by Selim et al. [65]. The objectives of their study were to examine the effects of GP (3, 6, and 9%) on lay rate, egg quality, yolk lipid composition, oxidative stability, and serum biochemistry. The researchers characterized the GP by evaluating the TPC, TFC, phenolic profile, and FA composition. They reported gallic, ferulic, chlorogenic, caffeic, and cinnamic acids among phenolic acids, and hesperidin, quercetin, and catechin among flavonoids. Regarding FAs, the prevalence of PUFAs (64.65%; *n*-3 PUFAs 1.78%; *n*-6 PUFAs 62.85%) was reported compared to SFAs (15.53%) and MUFAs (19.82%). Regarding the effect of experimental diets on egg yolk FAs, cholesterol, and triglyceride contents of laying hens, the researchers reported that modification of laying hens’ diets with GP up to 9% enriched egg yolk with beneficial *n*-3 PUFAs and reduced yolk cholesterol level compared to other treatment groups [65]. GP inclusion decreased both SFA/MUFA and SFA/PUFA ratios in the egg yolk. Dietary feeding enrichment (GP up to 90 g/kg) of laying hens increased egg yolk quality and oxidative stability without affecting laying hen health status. The results of these papers highlight the importance of a correct percentage of GP addition since, in many papers, the amount added is not sufficient to obtain the desired results.

#### 4.2.2. Miscellaneous (Pigs, Rabbits)

Pigs are one of the most important livestock animals in the world; therefore, it is important to improve the efficiency of pig production and also reduce antibiotic treatments. Some recent studies reported that reused agricultural by-products could reduce the cost and environmental problems linked to intensive breeding. In this context, Tian et al. [68] added GP (6%) to the finishing feed of pigs instead of wheat bran and analyzed the meat quality, immune performance, and intestines of pigs. The authors performed a targeted metabolomics profiling by UHPLC-MS/MS to determine AA and FA content. The experiment demonstrated that 6% supplementation did not cause differences in growth performance and carcass weight between the GP-treated and the control groups. The water loss, drip loss, and cooking loss of the treated group were significantly lower than those of the control group, while the content of *n*-6 PUFAs and alanine was significantly higher. An interesting aspect that has been investigated little in this research field is the analysis of the microbiota. From the analysis of the fecal microbiota of pigs, it emerged that the use of GP to fortify the feed reduced the relative abundance of Treponema and Streptococcus species, with positive implications on the immune performance and antioxidant capacity of fattening pigs [68]. Some studies reported that the change in microbiota could affect a pig’s growth, immune performance, and meat quality. Moreover, improving gut health could lead to reduced use of antibiotics [74]

Another interesting study is that of Ospina-Romero et al. [67]. They evaluated physiological variables, production performance, and carcass quality of fattening pigs fed a diet supplemented with ferulic acid (25 mg/kg) and GP meal (GPM, 2.5%) flour under heat stress conditions (suitable, mild, moderate, and severe zone). For each experimental diet, the chemical composition was determined with particular attention to the content of phenols, flavonoids, and the antioxidant capacity determined by three complementary assays (FRAP, ABTS, and DPPH methods). The effect of individual and combined supplementation (ferulic acid and GPM) on the productive performance of pigs did not lead to any significant effect on average daily weight gain and feed conversion. On the contrary, there was an interaction effect of ferulic acid and GPM on feed intake, which increased only if GP was present in the feed. Although no significant differences were found in mean daily weight gain, this parameter increased by 10% in GPM-treated animals, and an increase of 3 kg in fat mass was achieved compared to controls. The study showed that dietary supplementation with GPM flour in fattening pigs exposed to heat stress conditions improved feed intake and some carcass characteristics, modifying the degree of marbling and the relative weight of the liver. Although the physiological variables were not modified, GPM can be considered suitable as a zootechnical additive in the productive performance of pigs, even in conditions of environmental stress [67].

The process of obtaining GP and the storage method are two important steps in preserving its native characteristics. Despite that, only a few papers address this topic. The effects of supplementation of the diet of pigs with GP (20%) preserved in silage were evaluated by Giuliani et al. [70]. Moreover, the interaction between indoor and outdoor production systems on meat quality was also examined. The authors analyzed the chemical composition, cholesterol content, FA profile, shear force, texture profile, and sensory parameters. During cold storage, oxidative stability and objective color were also analyzed. The chemical composition of GP, TPC, anthocyanins, and FA profiles were also determined. The results did not show an interaction effect between the production and feeding system, and there were no significant differences in the proximal composition. The GP supplementation showed the lowest TBARS values during storage, i.e., less lipid oxidation in meats. However, the treatments with the GP inclusion showed the lowest TBARS values during storage, showing lower lipid oxidation in meats. The results showed no interaction between the FA profile and cholesterol levels in the produced meat. Cooking losses did not show significant differences. The GP silage does not alter the proximal composition, cholesterol content, FA profile, juiciness, and meat flavor. Moreover, GP silage improved pork quality, including tenderness, color intensity, and oxidative stability [70].

Silage produced from waste by-products of Greek olives, GP, and feta cheese was evaluated by Magklaras et al. [69] as a feed ingredient (0, 5, and 10%) for weaned pigs. The potential beneficial effects on performance, health, and balance of the intestinal microflora of pigs were evaluated, as well as a chemical, microbiological, and qualitative analysis of the meat (shoulder, thigh, and abdomen). The results indicated that the experimental silage had no negative effects on growth performance, intestinal function, microbial balance, and health characteristics of weaned pigs. The pH, fat, collagen, color, and protein contents did not differ between the meats of the different experimental groups. Furthermore, FA analysis of the meat showed that the n-6/n-3 ratio was improved. Regarding TPC and oxidative stability, it was highlighted that both parameters were higher in all parts of meats analyzed for the treatment containing 10% of by-products [69]. This study was well structured, but unfortunately, it was not very detailed regarding the chemical composition of pig meat. For example, it would have been interesting to characterize the FA profile of pig meat.

Some studies reported on GP supplementation of feed for RABBITS, monogastric animals. Rabbit meat is traditionally part of the Mediterranean diet, and its nutritional characteristics, including low fat and cholesterol content and a high percentage of PUFAs, are widely recognized [71,75]). Furthermore, based on the European Union trend, the challenge of the rabbit market is to minimize the use of medical products for their production. Interestingly, Bouzaida et al. [71] compared a medicated diet with a non-medicated control diet supplemented with 20% GP. They examined rabbit meat to evaluate changes in the production, FA composition, antioxidant properties, and shelf life. These authors were among the few who reported the chemical composition, FA composition, TPC, antioxidant capacity (DPPH), and reducing power (FRAP) of the control diet, GP diet, and GP by-product. An interesting aspect of the research was to investigate by HPLC-ESI-QTOF whether GP supplementation allowed the transfer of phenolic compounds to the meat. Among the different feed samples, 42 phenolic compounds were identified: 38 of which are present in the GP by-product, 14 compounds belonged to the flavanols (procyanidins and derivatives and catechin 3-O-gallate), 6 compounds correspond to the flavonols, 7 were anthocyanins, and 8 phenolic acids. With the exception of a few, most of the phenolic compounds present in the by-product were found in the GP diet. Bouzaida et al. [71] affirmed that other studies have reported that dietary phenolic compounds may not be detected in meat as they are modified, degraded, excreted, or retained in tissues [40,76]. Regarding production performance, rabbits fattened with GP supplementation, and those fed a commercial diet showed no significant differences in live weight assessed periodically, even if the feed conversion rate was higher and carcass weight and yield were lower for animals fed the GP diet than the control diet. Regarding fat percentage and FA composition of intramuscular fat, GP inclusion increased fat, n-3 PUFAs, PUFA/SFA ratio, and n-6/n-3 ratio, and decreased SFA percentage of Longissimus muscle backs. TPC and antioxidant properties were not significantly affected by dietary supplementation with GP. Regarding meat shelf life, the inclusion of GP reduced seemingly protein deterioration in rabbit meat, although no effect on lipid oxidation was found in minced hind leg meat stored for up to 6 days [71].

Differently from the previous studies, the research by Birolo et al. [72] evaluated the effect of dietary fortification (0.2% and 0.4%) with two high-tannin extracts (chestnut and GPE) on growth performance, nutrient digestibility, and meat quality in growing rabbits. The diet digestibility (dry matter and fiber fractions) changed significantly with the 0.4% supplementation level of chestnut and GP extracts. On the contrary, no significant effects on meat quality traits and oxidative stability were observed. Therefore, both high-tannin extracts can be safely used in growing rabbits at both tested percentages. The GP tannin content and composition accounted for a more favorable effect on nutrient digestibility than chestnut extracts. Further research details would have been helpful in deepening the results of this study.

Unlike other papers, Abdel-Rahman et al. [73] studied the effect of grape seed integration on some female pregnant rabbits at 14 and 21 gestation days and at kindling day. The basal diets were supplemented with hydrolyzable tannins (HI, 85% of polyphenols) at a dose of 1.5 g/kg or grape seeds extract (GSE) at a dose of 0.5 g/kg diet, and a combined mixture of both additives (HI + GSE, 0.5 g/kg). They reported that GSE contained phenolic compounds such as gallic acid, catechin, and procyanidin. Among the evaluated parameters, the levels of progesterone, plasma triglyceride, and cholesterol showed the highest value in the control compared to the other three treated groups. Furthermore, the treated groups had slightly lower MDA values and higher glutathione peroxidase 1 (GPX) activities than the control group. In the treated groups, milk production capacity increased compared to controls, particularly in the group fed the combination of both supplements (HI + GSE) [73].

### 4.3. Fish

Recently, some papers have been published regarding the GP integration in aquaculture farms to feed different kinds of fish. All around the world, aquatic systems are increasingly recognized for their multiple solutions to improve food security and nutrition and stimulate socio-economic development, especially for the many coastal and riparian communities, while maintaining a low environmental impact [77]. Table 3 shows research papers (2020–2024) focused on GP integration of fish diet.

In the paper of the research group of Mohammadi et al. [78], the effects of diets containing grape seed proanthocyanidin extract (GSPE: 200, 400, 600, 800, and 1500 mg/kg diet) on common carp (*Cyprinus carpio*) were studied. Fillet protein content, SOD, and glutathione peroxidase activity were significantly higher in the G200 group than in the control group. Dose-dependent effects of GSPE were observed even if the 1500 mg/kg level provided a reverse response. Recently, Mahmoodi et al. [79] fed common carp (*C. carpio*) with 5, 10, and 15% GP (G1, G2, and G3, respectively). They evaluated carp fillets’ survival index, growth, and chemical composition. Weight gain and specific growth rate (%) were significantly higher in the G3 compared to the G1 and G2 groups. Regarding the ash content, the 5% GP-treatment showed the lowest value (1.08%), while regarding crude lipids, the 5% GP-treatment had the highest content, and the G3 treatment was the lowest (6.03% vs. 3.90%). The results showed a significant difference in carbohydrate content, i.e., the control treatment had the highest carbohydrate content, and the 10% treatment had the lowest (0.61% vs. 1.28%). For estimated energy values of carp fillets, the 5% grape pomace treatment showed the highest caloric content, while the 15% treatment had the lowest caloric content [79]. Among the weaknesses of these studies, the following can be mentioned: the lack of a detailed FA composition of the fillets, the amount of individual AAs, the TPC, and the phenolic profile. Recently, meat quality, microbiota, and oxidative status of carp (*C. carpio*) were also studied by the research group of Barbacariu et al. [80]. The study revealed that most growth parameters were not significantly affected by the GP diet, except for a notable difference in liver size relative to body weight. Significant differences were found in the biochemical analysis of carp meat. The GP inclusion modulated oxidative status and lipid peroxidation pathways in carp, enhancing their health, especially their antioxidant defenses. Furthermore, microbiological examination of the carp intestinal content showed that GP supplementation influenced some microbial parameters (aerobic germs and Enterobacteriaceae) [80]. Tarricone et al. [81] evaluated the dietary effect of the inclusion of a polyphenol-based extract derived from GP (100 or 200 mg/kg) on fillets of aqua-cultured sea bass (*Dicentrarchus labrax*). After the experimental period, the quality parameters of the fish meat, including the FA profile, were evaluated. Regarding growth parameters, no differences were highlighted between the experimental treatments, while the overall consistency of fish fillets improved following the inclusion of polyphenols in the diets. Furthermore, the polyphenol-rich red grape extract lowered the total lipid content of the fish, the SFA concentration, and the AI while increasing the PUFA content. In both treatments, a lower concentration of MDA was detected, thus confirming the antioxidant effect of the polyphenol-rich extract and its effectiveness in preventing lipid oxidation [81].

Last but not least, the experimental works of Martínez-Antequera et al. [82,83] should be mentioned. In the first article, the potential benefits of including red GP or lees (100 g/kg) as functional ingredients in aquaculture feeds for juveniles of *Liza aurata* were evaluated [82]. The study was designed as two different experiments focused on evaluating the effects of supplementation on metabolism and the putative protective effect against stress induced by moderate hypoxia. Regarding the growth and biometric parameters evaluated, it emerged that the group that integrated GP presented significantly higher values of final body mass. Plasma and liver metabolites revealed significantly lower plasma lactate levels in the two experimental groups compared to the control. However, no significant differences were observed for other circulating parameters. In addition, significantly lower liver glycogen values were observed in fish receiving the GP diet compared to the grape lees-fed group. Regarding immune status, lysozyme activity in mucus was significantly increased in the GP group compared to the other experimental groups. The study also found that supplementation with each of the two grape by-products provided antioxidant protection against the challenge of medium hypoxia as it led to lower cortisol levels and changes in the activities of some enzymes involved in reactive oxygen species (ROS) control, especially in the GP-supplemented group [82]. In a successive study, the same research group evaluated the impact of GP on the diet of young European seabass (*Dicentrarchus labrax*) [83]. It should not be underestimated that intensive farming systems in aquaculture are often used and that these cause an increase in environmental stressors, facilitating the appearance of pathologies in fish. Potential changes in metabolism, immunological and oxidative status, and functionality of the intestinal microbiota were evaluated, as well as the potential protective effect against oxidation in fish fillets and the preservative effect of GP (0.4%) in the feed. The selected dosage, although lower than that used in previous studies involving aquaculture species, was chosen to assess the impact of GP at a level more consistent with those used for similar compounds in commonly used aquaculture feeds. The total antioxidant capacity (TAC) did not show any significant effect of GP added to the standard diet at the two storage temperatures (4 and 25 °C) (Figure 2D). As regards the growth and biometric parameters of the sea bass, no significant differences were highlighted between the two experimental groups except for a greater length of the intestine in the GP-treated group. Regarding the biodiversity of the microbiota of the experimental groups, a notable influence of GP was observed on the microbial profile, which was characterized by a lower number of species compared to the controls. Biochemical and immunological parameters evaluated in plasma, muscle, liver, and skin mucus revealed significantly lower hematocrit and plasma cortisol values in the GP-supplemented group, as well as reduced glycogen levels in the liver and muscle. For the evaluation of the preservation of the fillets, it was found that after 2 days of storage, the samples of fish fillets fed with GP showed significantly lower levels of ROS compared to the control group. Interestingly, fish fed the control diet maintained constant levels of lipid oxidation of the fillet during the 6 days of storage, while the fish fed with GP showed a significant decrease in the oxidative status [83].

A particular study was carried out by Bullon et al. [84]. Abalone (*Haliotis iris*) represents a valuable export product in New Zealand, and due to the high cost of the feed, insect meal and GP are potential candidates to be included in aquafeeds. The inclusion of these products (30%) did not affect the protein proportions of abalone tissues, but differences in the proportions of carbohydrates and lipids were found. The inclusion of insect meal and GP significantly affected the levels of some L-amino acids (histidine, methionine, and phenylalanine) and FAs (oleic, linoleaidic, palmitic, palmitoleic, and α-linolenic acids), suggesting a correlation between the feed source and FA tissue composition [85].

All these recent works on aquaculture provide promising results for the future optimization of diets, including more affordable and sustainable alternative ingredients.

## 5. Chemometrics Approach

As regulatory requirements for food quality are increasing, practical approaches for the quality assessment of food of animal origin food represent a promising challenge. At present, several analytical methods are often combined with chemometrics to discriminate the authenticity of products. Principal component analysis (PCA) is the most commonly used algorithm to reduce the dimensionality of data [85].

**Figure 4:**
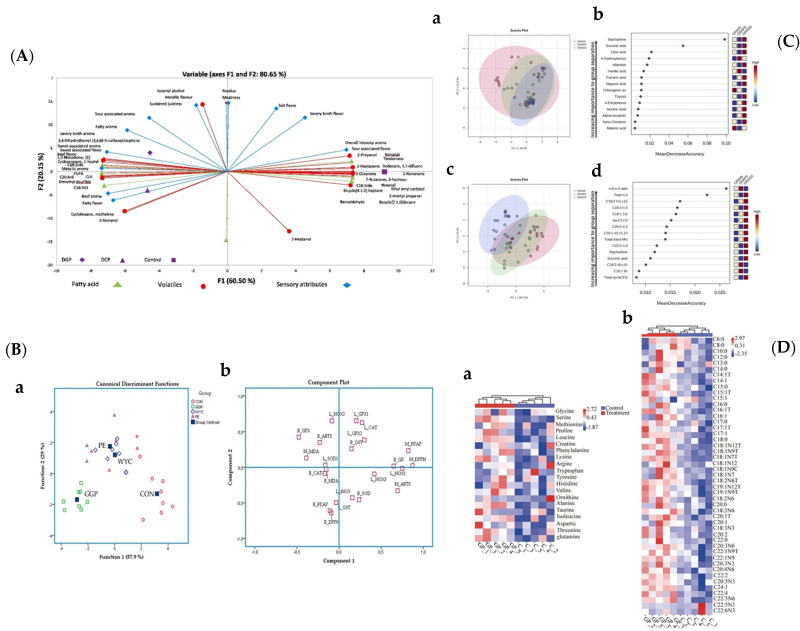
(**A**) Variable plot obtained from principal component analysis illustrating relationships between PUFAs, volatile, and sensory profiles of beef fed control, DCP, or DGP diets [39]. Reproduced with permission, Copyright 2020, Elsevier. (**B**) (**a**). Discriminant plots separating the four dietary treatments according to pooled data of the liver’s relative transcript levels and blood plasma and breast muscle antioxidant indicators. (**b**). Principal component analyses were applied on liver relative transcript levels, blood plasma, and breast muscle antioxidant indicators [57] (**C**) (**a**). Principal component analysis (PCA) plot using phytochemicals only showing separation and clusters based on finishing diet with some overlaps. (**b**). Random forest (RF) variable importance plot showing main phytochemicals capable of discriminating beef based on finishing diet. (**c**). PCA plot using phytochemicals and fatty acids showing three clusters based on finishing diets. (**d**). RF variable importance plot showing main fatty acids and phytochemicals capable of discriminating beef based on finishing diet. For RF plots, the y-axis represents phytochemicals in order of importance for group classification (from top to bottom). The x-axis shows a mean decrease in accuracy, with a higher value indicating the importance of that phytochemical in predicting groups [40]. (**D**) (**a**). Heatmap of amino acid in the control and treatment groups. (**b**). Heatmap of fatty acid in the control and treatment groups [60].

Figure 4 (A-D) show the chemometric approach adopted by some authors. For example, Tayengwa et al. [39] used PCA to evaluate the relationships between FA, volatile, and sensory profiles of meat from cattle fed a control diet and enriched in dried grape pomace (DGP) or dried citrus pulp (DCP). The control diet was clustered with C18:1 *n*-9, aldehydes, ketones, alcohols, sour-associated flavor, overall intensity aroma, and tenderness. DGP-fed beef was closely clustered with C18:2 *n*-6, 3,4-dihydrothienyl (3,4,b)-5-carboxythiophene, sustained juiciness, flavor (sweet associated, beef and metallic) and aroma (metallic, sweet associated, fatty and savory broth) attributes. The DCP was closely clustered with C20:4*n*-6, C18:3*n*-3, dimethyl disulfide, beef aroma, fatty aroma, cyclohexane, methylene, and 2-nonanol in the lower left quadrant (Figure 4A). Interestingly, the presence of aldehydes (i.e., hexanal and nonanal) in beef from the control diet compared to the beef from the DCP and DGP diets is consistent, indicating a greater degree of lipid oxidation. The protective effects of antioxidants in DCP and DGP diets were also found to extend beyond lipids, as some aldehydes (i.e.*,* benzaldehyde, 2-methyl-propanal, and 2-methylbutanal) in control-fed beef are likely products of amino acids (i.e., valine, isoleucine and leucine) degradation.

PCA was also used by Mavrommatis et al. [57]. They applied this approach to the pooled data of liver relative expression levels of selected genes and the antioxidant indicators of both blood plasma and breast muscles of broiler chickens to reduce the data’s dimensionality and underline the relationships between the variables. Twenty-two variables, including FRAP, ABTS, and DPPH values of blood and breast, as well as MDA and antioxidant enzymes, were entered to create a model to distinguish the samples. A clear separation between GGP and the other groups (CON, WYC, PE) is shown (Figure 4B). Moreover, to the same data, they also applied a discriminant analysis (DA). This approach allocated the PE and WYC groups together since both their polyphenolic compositions and levels were quite comparable. On the contrary, the GGP group was mapped away due to the altered response to dietary treatment attributed to PUFAs and polyphenol levels.

In another paper concerning the fortification of feed for beef, Krusinski et al. [40] applied PCA and random forest (RF) analysis (Figure 4C). Initially, they reported the PCA plot separation, with overlaps, between beef from the three different finishing systems (GRAIN, GRAPE, GRASS), showing a 30% variation along principal component 1 (PC1). In this first approach, they used only phytochemical compound data, but when the authors included FA data, PCA showed separation without overlaps, showing a 29.60% variation along PC1. Interestingly, in the Random Forest analysis, the *n*-6/*n*-3 ratio and total *n-*3 PUFAs were the two most discriminating factors, although stachydrine and succinic acid remained among the most important phytochemicals to separate beef by diet, followed by citric acid, 4-hydroxybenzoic acid, allantoin, and vanillic acid. These results are very intriguing since they highlighted the importance of metabolomics data in evaluating the impact of different feeds. Moreover, the inclusion of the FA composition data in the dataset improved the discrimination, giving these nutrients an important role linked to the reduction in the out-of-bag error rate.

Tian et al. [68] highlighted that GP improved meat quality in finishing pigs using a chemometric approach. PCA showed differences in amino acid and FA content (Figure 4D). Alanine in the treatment group was significantly higher than the control group. In addition, the contents of C14:1 *t*, C18:0, C18:2 *n-*6, and C20:4 *n-*6 in the treatment group were significantly higher than those in the control group. The TAC and the activity of SOD in the treatment group was significantly higher than that in the control group. Meanwhile, the MDA content and ROS activity in the treatment group were significantly lower than those in the control group. Based on these results, the authors affirmed that pigs’ gut health was increased, as also confirmed by other studies showing that a diet with phenolic compounds could attenuate oxidative stress in piglets [68].

Based on the topic of this review, it should be pointed out that only in a few articles was a chemometric approach applied to evaluate the relationships between the analyzed parameters (variables) and to discriminate animals fed with one type of feed rather than another.

## 6. Conclusions

Nowadays, food quality issues have gained great attention from researchers, food processors, and consumers. All around the world, improving the food quality and shelf life of animal-based foods is a critical issue for the agro-food industry to protect human health and minimize the use of medical products in animal production. Moreover, it is desirable to counteract damage to the ecosystem by increasing a circular economy policy. The GP, a by-product generated by the winery industry, has many phytochemicals with interesting biological activity, such as phenols, unsaturated FAs, and fiber. The valorization of these fruit by-products as animal feed ingredients and natural sources of meat preservatives has the potential to improve the sustainability of the feed and meat industries and the shelf life of foods, together with the reduction in waste management costs incurred by the fruit processing industries (i.e., vinery). Thus, the environmental impact of livestock production is reduced, contributing to the sustainability of animal production systems and covering the demand for food of animal origin, which affects the physical and mental health of humans.

The novelty of this review can be observed through the emphasized influence of GP addition on different food commodities produced by various types of animals. This review confirmed that GP can be successfully used as a fortifying ingredient in animal feed since the beneficial nutritional profile of GP can be readily transferred to the fortified product, even if some considerations are needed. It can be affirmed that generally, some of the nutritional characteristics of meat, milk, eggs, and fish are improved after feed fortification (i.e., phenol and FA profile), contributing to human access to a healthier diet, due to the high content of antioxidants and healthy FAs. Phenolic compounds showed a promising role in increasing the antioxidant potential of feed, even if it should not be forgotten that phenol content and profile are strongly related to the cultivar and geographical origin of the grape.

Generally, the effects of GP on the FA profile showed positive effects on improving the nutritional composition of fat in foods such as milk. In particular, there was a reduction in SFAs, with an increase in MUFAs and PUFAs. Another interesting aspect concerns the sensory properties of the final product since they may be differently and adversely affected by GP addition to feed. In some cases, modifications to the fortified product are necessary because sensory attributes play a major role in the acceptance of a product by a greater number of consumers. In some cases, one of the main drawbacks of fortification in plant food products was the undesirable change toward a darker-brownish color, while in meat products, this change brought a positive response among the sensory panelists. Sensory attributes of the final product, potentially subject to changes after GP inclusion, play a major role in consumer acceptance. For this reason, all studies concerning this topic should provide these data.

Moreover, this review demonstrates that GP has benefits in the productive and growth performances of livestock when included in the diet. The intestinal health and well-being of animals, as well as the quality of the final product, were generally improved after GP feeding. Only a few studies are concerned with the storage process of the final food, even if this aspect is important for a correct evaluation of the shelf life, especially for long-life products. It must be considered that MUFAs and even more PUFAs are prone to oxidation and, therefore, modify the shelf life of animal foods.

In some cases, the results of the research activity confirmed the positive effect of GP feeding on the fattening parameters of animals. Regarding feed intake and digestibility, in some studies, a reduction in these parameters was highlighted, probably due to the increase in fiber. In some cases, the supplementation of GP is still controversial, and further research is needed. For example, more studies using different amounts of GP over longer periods would be useful, and a deep characterization of the phenol fraction of GP and final food is desirable.

Another intriguing aspect regards the processing techniques for transforming GP in feed for animal nutrition. They are necessary to increase the nutritional value, digestibility, and feeding efficiency, as well as to remove non-edible components from GP. For this reason, further research in this area is certainly needed to test the bioactivity of various kinds of feed after different technological treatments and to evaluate their impact on final production.

Finally, it is desirable to establish an interdisciplinary approach between veterinary knowledge, food technologists, and chemical–analytical experts, also supported by chemometric methods to optimize and increase the use of GP as a valuable ingredient for feed use.

## Figures and Tables

**Table 1:** Examples of research papers (2020–2024) focused on grape pomace integration in the diet of ruminants.

Ruminants	Animals Details	Grape and GP Details	GP Characterization (Details on Phenols)	Products	Main Evaluation Items	Remarks	Reference
**Meat**							
	Jersey × Holstein (*n* = 24)	HGP 58% DM Ensiled GP	HGP (on DM): TPC 3.19% Free phenols 0.43% TTC 2.77% NT 0.42%	Strip loins (LL) Top-round SM muscles	Carcass data Retail color Lipid oxidation On steaks: FA analysis Tenderness	↑ L* and b* values: HGP LL steaks HGP LL and SM steaks: ↑ total PUFAs, C18:2*n*-6, C18:2 *c*9*t*11, CLAs ↓ lipid oxidation (MDA)	[38]
	Angus (*n* = 24) 7 months aged	150 g/kg DM DGP	TPC 177.3 g GAE/kg DM TTC 104.2 g GAE/kg DM CT 33.3% LE	LT and LL muscles	Intramuscular FA analysis Volatile compound analysis of fresh raw meat Sensory analysis	↑ *n*-6 PUFAs (C18:2*n*-6, C20:4*n*-6) ↑ *n*-3 PUFAs (C18:3*n*-3) ↑ CLAs ↓ C18:1*n*-9, total aldehydes, ketones, and alcohols	[39]
	Red Angus (*n* = 72) 14–20 months aged	5% DM GSE Dried grape pulp	Gross composition	Steaks	Proximate analysis FA analysis Vitamin E Mineral analysis Phenolic profile	↑ *n*-6 PUFAs ↑ zinc concentration ↑ vitamin E concentration	[40]
Lambs/ewes	Texel lambs (*n* = 24, castrated males) 10–12 months aged	25, 37.5, and 50 kg/100 kg of diet - Bordeaux variety	Gross composition On DM: TPC 8.95–15.06 mg GAE/g TTC 0.69–1.52 mg/g	LT and LL muscles (12th and 13th ribs) Blood Liver	Carcass characteristics Blood parameters Liver parameters	↓ carcass cover fat ↑ TPC	[41]
	Lambs (*n* = 30) 25–30 days aged	10% GP DM - Red variety	TPC 73.36 mg GAE/g TAC 496.12 μmol TEAC/g	LD muscle	Meat color Drip loss Cooking loss Chemical composition FA profile Lipid oxidation Volatile compounds	↑ % stearic, vaccenic, rumenic acids ↑ nonanal and 1-octen-3-ol ↓ hexanal	[42]
	Texel lambs (*n* = 24, non-castrated) 100 days aged	150, 300, and 500 g/Kg DM silage - Merlot variety	TPC 42.3 vs. 18.3 g/kg TTC 22.8 g vs. 1.8 g/kg CT 14.1 vs. 1.8 g/kg	LT and LL muscles	Performance and gross margin Carcass and meat quality FA profile	↓ daily weight gain ↑ gross margin ↑ PUFAs, *n*-6 PUFAs ↑ n6/n3 ratio ↑ 18:2 *n-*6 (LT and LL muscle) ↓ 16:0 (LT and LL muscle)	[43]
	Han lambs (*n* = 28, males) 3–4 months aged	6% DM - Grape seeds from China	TTC 24.2 g/kg DM	LT muscle	Meat quality FA analysis AA analysis	↑ meat redness ↓ methionine in meat ↓ SFAs ↑ EPA and DHA ↓ TI	[44]
	Churra ewes (*n* = 48) 3 and 5 years aged + their suckling lambs	5, 10% DM - Red grape variety	Gross composition Extractable polyphenols 42.8 g/kg CT 54.6 g/kg Anthocyanins 4.10 g/kg	LT and LL muscles	Microbiological analysis Color measurement Shelf life analysis	↓ metmyoglobin % ↓ lipid oxidation (TBARS) ↑ sensory perception	[45]
**Milk/dairy products**							
Lambs/ewes	Crossbreed dairy ewes (*n* = 46)	10% red GP (flour) - Montepulciano d’Abruzzo variety	TPC 2.24 mg GAE/g TAC 502.59 µmol TEAC/g	Milk	Chemical analysis TPC ABTS assay FA profile Protein content AA profile	↑ MUFAs ↑ DIC18 ↓ MCSFAs ↓ DIC14	[46]
	Assaf ewes (*n*= 46)	10% GP - Red grape variety	-	WBR and WPR cheese-making Ricotta, after 1 (T1) and 5 (T5) days of ripening at 4 °C	Chemical analysis Color measurement TPC ABTS assay FA profile Whey protein Volatile compounds	WBR: ↓ L* and a* values ricotta T1: ↑ TPC ↓ nonanal ↑ octanoic acid ricotta T2: ↓ hexanal	[47]
	Crossbred ewes (*n* = 46)	10% GP Red grape variety	Phenol profile	Milk	Phenolic profile Antioxidant status Inflammatory status Milk gelatinase activity Zymographic analysis Metalloproteinases Molecular docking	↓ phenolic acids and flavones ↑ flavanols and flavonols ↓ MMP-9 activity	[48]
Goats	Camosciata delle Alpi (*n* = 22)	150 g/d dried GP - Red Barbera grape variety	TPC 27.0 mg GAE/g DM CT 10.3 mg LE/g DM	Milk	Yield Composition FA profile	↓ rumenic acid	[49]
	French Alpine (*n* = 24) 5 years aged	GSC5: 5% (75 g/d) GSC10: 10% (150 g/d) - Cabernet franc variety	On kg DM: TPC 418.22–812.6 mg Anthocyanins 155.80–240.46 mg	Milk	Hematological parameters Blood enzyme activities Antioxidant status	GSC10: ↑ SOD and GR activity ↓ glucose ↓ somatic cell count	[50]

a*, red-green axis; AAs, amino acids; ABTS, 2,2′-azino-bis(3-ethylbenzothiazoline-6-sulfonic acid; CLA, conjugated linoleic acid; CON, control; CTs, condensed tannins; DGP, dried grape pomace; DHA, docosahexaenoic acid; DIC14, desaturation index 14; DIC18, desaturation index 18; DM, dry matter; EPA, eicosapentaenoic acid; FAs, fatty acids; GP, grape pomace; GR, glutathione reductase; GSC, grape seed cake; HGP, finishing diet containing 58% grape pomace; L*, lightness; LD, *Longissimus dorsi*; LE, leucocyanidin equivalent; LL*, Longissimus lumborum*; LT, *Longissimus thoracis*; MCSFAs, medium-chain saturated fatty acids; MDA, malondialdehyde; MMP-9, matrix metalloproteinase-9; MUFAs, monounsaturated fatty acids; NT, non-tannins; PUFAs, polyunsaturated fatty acids; SFAs, saturated fatty acids; SOD, superoxide dismutase; SM, semimembranosus; TAC, total antioxidant capacity; TBARSs, thiobarbituric acid reactive substances; TEAC, Trolox equivalent antioxidant capacity; TI, thrombogenic index; TPC, total phenol content; TTC, total tannin content; WBR, whey before ricotta; WPR, whey post ricotta. -, data not reported; ↑ indicates the promotion trend; and ↓ indicates the downward trend.

**Table 2:** Examples of research papers (2020–2024) focused on grape pomace integration in the diet of non-ruminants.

Non-Ruminants	Animal Details	Grape and GP Details	GP Characterization (Details on Phenols)	Products	Main Evaluation Items	Remarks	Reference
Broiler chickens	Ross-308 (*n* = 50, mixed sex) 42 days aged	1, 2, 3% Alibernet variety	Gross composition	Breast meat Thigh meat	MDA analysis on the 1st, 3rd, and 5th day of storage	Any significant difference No negative effects	[55]
	Ross-308 (*n* = 80; mixed sex)	1, 2, 3 100/kg FM - red GP Alibernet variety	Gross composition	Breast meat Thigh meat	AA profile FA profile	Breast muscle: ↑ MUFAs (C18:1*n-*9) Thigh muscle: Significant differences in some AAs	[56]
	Aviagen Ross-308 (*n* = 240) 1 day aged	GGP 25 g/kg PE 1 g/kg - Native Greek Assyriko variety	On 25 g GGP DW: TPC 193.2 mg GAE TFC 50.3 mg QE TTC 319.5 mg EE Phenol/FA profile	Breast muscle Blood	Chemical analysis Antioxidant enzyme activities Oxidative stability	PE-fed: ↓ NOX2 and SOD ↑ TAC (FRAP assay) ↓ MDA values	[57]
	Ross-308 (*n* = 216, male) 1 day aged	Silage-5% Silage-10%	Gross composition		Chemical analysis Color analysis Oxidative stability FA profile	Silage-10% ↓ MDA ↑ thigh meat oxidative stability	[58]
	Ross-308 (*n* = 750, male) 2 weeks aged	30 g/kg - Red GP powder + AVG	AVG, phenolic compounds (1%)		Growth performance Blood examination Carcass characteristics and internal organs	↑ weight gain, feed conversion ratio, finisher weight	[59]
	Ross-508 (*n* = 112, male)	2.5, 5, 7% DGP Red grape (*Vitis vinifera* L.)	Gross composition TPC 15.87 mg GAE/g FA profile	Breast meat	Physicochemical analysis FA profile Lipid oxidation Volatile compounds	5 and 7% DGP: ↑ PUFAs (linoleic acid) ↓ SFAs ↓ lipid oxidation ↓ volatile aldehydes	[60]
Geese	Czech goose breed (*n* = 20) 28 days aged	1% - White wine variety (Pinot Gris)	TPC 27.38 mg GAE/g TPA 13.27 mg CAE/g TFC 0.12 mg QE/g TAC 9.17 mg TE/g	Abdominal fat	Fattening parameters FA profile	↑ liver weight ↑ MUFAs (oleic acid) ↓ PUFAs ↓ SFAs	[61]
Quail chicks	Japanese (*n* = 288, male) 15 days aged	3% GSP or GSM	Gross composition Phenol profile FA profile	Thigh muscle Breast muscle	Performance Blood biochemistry FA profile Antioxidant status HSP70 gene expression	3% GSM: ↓ HSP70 levels Diet containing 3% GSP or GSM was not effective in overcoming oxidative stress	[62]
Layer chickens	Hy-Line W36 (*n* = 75) 50 weeks aged	GP: 30 or 60 g/kg GE: 0.5 or 1.0 g/kg - Spanish red grapes (Cencibel variety)	GP-TPC 4.91–7.42 g GAE/kg GE-TPC 2.65–2.86 g GAE/kg	Egg Egg after 4 months of storage	Hen performance Egg quality Chemical analyses (TPC, FA profile, oxidation assessment)	For 60 g/kg GP: ↓ SFAs, MUFAs (yolks) ↑ PUFAs (stored eggs) ↓ MDA values	[63]
	Hy-Line W36 White Leghorn (*n* = 576) 57 weeks aged	0 or 5% DGP - Iranian red/white grapes	Gross composition	Egg	Egg quality Antioxidant status Yolk weight, color, cholesterol	↓ feed intake ↓ egg mass ↑ egg yolk color and trait (5% DGP)	[64]
	Lohman Brown Lite (*n* = 200) 35 weeks aged	30, 60, 90 g/kg - Egypt red grapes (*Vitis vinifera* L.)	On DW: TPC 28.63 mg GAE/g TFC 8.42 mg CE/g	Egg	Egg quality Oxidation stability FA profile Serum biochemistry	↑ egg production ↑ egg weight ↑ antioxidant capacity	[65]
	ISA White and ISA Brown (*n* = 30, each)	50 g/kg	2.10 g GAE/100 g DW Phenolic profile	Egg	Egg quality Chemical analysis (vitamins A and E, phenolic compounds)	↑ yolk color score ↑ gallic acid	[66]
Pigs	Yorkshire × Duroc male (*n* = 40)	2.5% GPM	TPC 20.81 mg GAE/g TFC 11.30 mg CE/g HT 3.34 mg GAE/g CT 0.8 mg CE/g TAC 104.7μM TE/g DPPH 114.8 μM TE/g Phenol profile	Meat	Blood metabolites Hormonal levels Hematological parameters Biochemical parameters	↓ marbling ↑ loin area ↑ liver weight	[67]
	Fellow (Guanzhong Black Pig × Landrace) (*n* = 24, male)	6%	-	Meat (LT)	Meat quality AA and FA contents Inflammatory cytokines Antioxidant indices Microbiota	↓ water loss, IL-1β, DAO, ROS, MDA values ↑ IgA, IgG, IgM, CAT, TAC, SOD, IFN-γ	[68]
	Crossbreed weaned (1/4 Large White × 1/4 Landrace × 1/2 Duroc) (*n* = 45) 34 days aged	5% or 10%	Gross composition	Meat	Chemical/color analysis Oxidative stability Microbiological analysis FA profile	↑ oxidative stability ↑ *n*-6/*n*-3 ↑ ileum and cecum microflora populations	[69]
	50% Large White × 50% Landrace (*n* = 24, each group, mixed sex)	20% - Brazil red grapes (*Vitis vinifera*, Bordeaux variety)	TPC 12.92 mg GAE/g Anthocyanins 38.34 mg/g malvidine-3-glycoside	Meat (LT)	Chemical analysis FA profile Cholesterol (meat) Lipid oxidation Color analysis Texture profile Sensory analyses	↑ quality of the meat	[70]
Rabbits	New Zealand white (*n* = 36) 35 days aged	20% - Spain grapes (Grenache variety)	Gross composition Phenol profile FA profile On 100 g DW: TPC 491 mg GAE DPPH 13,032 μmol TE FRAP 3657 μmol TE	Meat (LD)	FA profile Phenol profile TPC, TAC (DPPH assay), (FRAP assay pH MDA value	↑ linolenic acid ↓ α-linolenic acid ↑ *n*-6/*n*-3 ratio ↓ SFAs ↑ PUFA/SFA ratio ↑AI and TI	[71]
	Crossbreed (*n* = 270, mixed sex) 30 days aged	0.2 or 0.4% GPE	-	Meat (LL) Hind leg	Growth performance Nutrient digestibility Meat quality	↑ DM digestibility ↑ fiber fractions ↑ gross energy	[72]
	New Zealand White (*n* = 36, female) 8 months aged	HT 1.5 g/kg GSE 0.5 g/kg HT + GSE (0.5 g/kg)	Gross composition	Milk (kindling)	Blood analysis Does/litter performance TAC	↑ TAC ↑ GPX activities ↓ MDA values	[73]

AAs, amino acids; AI, atherogenicity index; AVG, aloe vera gel; CAT, catalase; CTs, condensed tannins; DAO, D-amino acid oxidase; DGP, dried grape pomace; DM, dry matter; DPPH, (2,2-diphenyl-1-picrylhydrazyl); DTP, dried tomato pomace; DW, dry weight; EEs, epicatechin equivalents; FAs, fatty acids; FM, fresh matter; FRAP, ferric ion reducing antioxidant power; GAEs, gallic acid equivalents; GE, grape extract; GGP, ground grape pomace; GP, grape pomace; GPE, grape pomace extract; GPM, grape pomace meal; GPX, glutathione peroxidase; GSE, grape seed extract; GSM, grape seed meal; GSP, grape seed powder; HSP70, heat shock protein 70; HTs, hydrolysable tannins; IFN-γ, interferon gamma; IL-1β, Interleukin-1 beta; LD, *Longissimus dorsi*; LL, *Longissimus lumborum*; LT, *Longissimus thoracis*; MDA, malondialdehyde; MUFAs, monounsaturated fatty acids; NOX, NADPH oxidase; PE, grape stem extract; PUFAs, polyunsaturated fatty acids; QEs, quercetin equivalents; ROS, reactive oxygen species; SFAs, saturated fatty acids; SOD, superoxide dismutase; TAC, total antioxidant capacity; TEs, Trolox equivalents; TEAC, Trolox equivalent antioxidant capacity; TFC, total flavonoid content; TI, thrombogenicity index; TPAs, total phenolic acids; TPC, total phenol content; TTC, total tannin content; TVB-N, total volatile basic nitrogen; WYC, wine lees extract; -, data not reported; ↑ indicates the promotion trend; and ↓ indicates the downward trend.

**Table 3:** Examples of research papers (2020–2024) focused on grape pomace integration in the diet of fish.

Animal Details	Grape and GP Details	GP Characterization (Details on Phenols)	Products	Main Evaluation Items	Remarks	Reference
Common carp (*Cyprinus carpio*) (*n* = 180)	200, 400, 600, 800, and 1500 mg/kg - GSPE: containing 95% proanthocyanidins	95% proanthocyanidins	Serum Fillet	Growth performance Biochemical parameters Liver enzyme activity Fillet proximate composition	↑ WG, SGR ↑ SOD, GPX ↓ serum glucose, cholesterol, triglyceride	[78]
Carp (*Cyprinus carpio*) (*n* = 200)	5, 10, or 15% (*w*/*w*) - Grapes (*Vitis vinifera* L.) Pellet	-	Fillet	Growth and survival indices Chemical composition Physicochemical parameters	At 15%: ↑ growth indices ↑ weight of fish ↑ survival rate	[79]
Carp (*Cyprinus carpio*) (*n* = 180)	5 or 10%	Chemical composition TPC 83.07 mg/g DW TFC 13.53 mg/g DW TAC (DPPH % inhibition) 81.87%	Meat Blood Microbiota	Growth indices Proximate composition Blood parameters Biochemical parameters Microbiological exam	At 10%: ↓ SOD ↑ CAT ↑ GSH (muscle tissue) ↓ MDA ↑ MPV	[80]
juvenile sea bass (*Dicentrarchus labrax*) (*n* = 180)	Red grape polyphenol extract (GPE): 100 (GPE 100) or 200 mg/kg (GPE 200) - Red grape Nero di Troia variety	Proanthocyanidins (101.8%) and catechins plus epicatechin (10.37%)	Fillet	Growth parameters pH Color Textural parameters Chemical analysis FA analysis	Both GPE diets: ↓ red (a*) index ↓ yellow (b*) index ↓ fillet hardness ↓ total lipid content ↑ PUFA content ↓ MDA values	[81]
Juvenile golden gray mullet (*Liza aurata*)	GP or WL (liquid form) 100 g/kg	Proximate composition	Skin mucus Blood Tissues	Growth performance Biochemical parameters Immunological parameters Microbiota Oxidative status	↑ growth performance ↑ feed efficiency - GP: ↑ immune status	[82]
Juvenile seabass (*Dicentrarchus labrax*) (*n* = 120)	0.4% - Red wine Spanish GP	-	Fillet	Growth performance Biometric parameters Gut microbiota Biochemical and immunological parameters Oxidative status Fillet quality during storage	Potential to prevent the oxidation in the absence of other preservatives	[83]
Commercial abalone (*Halioris iris*)	FG or FIG (30 g/100 g diet)	-	Tissues Feces	Growth parameters Proximate analyses (animal soft tissues and fecal samples)	With FIG: ↓ L-histidine, glycine, L-methionine, L-phenylalanine levels ↑palmitic, palmitoleic, stearic, oleic, α-linolenic, arachidonic acids	[84]

a*, red-green axis; b*, yellow/blue axis; DW, dry weight; FIG, fish meal + insect meal + GP; FG, fish meal + GP; GAEs, gallic acid equivalents; GP, grape pomace; GPE, grape pomace extract; GPX, glutathione peroxidase; GSPE; grape seed proanthocyanidin extract; MDA, malondialdehyde; MPV, mean platelet volume; TAC, total antioxidant capacity; TFC, total flavonoid content; TPC, total phenol content; PUFAs, polyunsaturated fatty acids; SGR, specific growth rate; SOD, superoxide dismutase; TFC, total flavonoid content; TPC, total phenolic content; WG, weight gain; WL, wine lees; -, data not reported; ↑ indicates the promotion trend; and ↓ indicates the downward trend.

## Data Availability

No new data were created or analyzed in this study. Data sharing is not applicable to this article.

## List of Abbreviations

AAs, amino acids; ABTS, 2,2′-azino-bis(3-ethylbenzothiazoline-6-sulfonic acid; AI, atherogenicity index; AOA, antioxidant activity; AVG, aloe vera gel; CAT, catalase; CLA, conjugated linoleic acid; CON, control; CTs, condensed tannins; DAO, D-amino acid oxidase; DCP, dried citrus pulp; DGP, dried grape pomace; DHA, docosahexaenoic acid; DIC14, desaturation index 14; DIC18, desaturation index 18; DM, dry matter; DPPH, (2,2-diphenyl-1-picrylhydrazyl); DTP, dried tomato pomace; DW, dry weight; EEs, epicatechin equivalents; EPA, eicosapentaenoic acid; FAs, fatty acids; FM, fresh matter; FRAP, ferric ion reducing antioxidant power; GAEs, gallic acid equivalents; GC/MS, gas chromatography–mass spectrometry; GE, grape extract; GGP, ground grape pomace; GP, grape pomace; GPE, grape pomace extract; GPM, grape pomace meal; GPX, glutathione peroxidase; GR, glutathione reductase; GRAIN, diet containing mixed cereals; GRASS, pasture grass; GRAPE, diet containing mixed cereals and 5% grape pomace; GSC, grape seed cake; GSE, grape seed extract; GSM, grape seed meal; GSP, grape seed powder; HGP, finishing diet containing 58% grape pomace; IFN-γ, interferon gamma; IL-1β, Interleukin-1 beta; HSP70, heat shock protein 70; HTs, hydrolysable tannins; LCSFAs, long-chain saturated fatty acids; LD, Longissimus dorsi; LE, leucocyanidin equivalent; LL, Longissimus lumborum; LT, Longissimus thoracis; MCSFAs, medium-chain saturated fatty acids; MDA, malondialdehyde; MMP-9, matrix metalloproteinase-9; MUFAs, monounsaturated fatty acids; NOX, NADPH oxidase; NTs, non-tannins; PE, grape stem extract; PUFAs, polyunsaturated fatty acids; QEs, quercetin equivalents; ROS, reactive oxygen species; SCSFAs, short-chain saturated fatty acids; SFAs, saturated fatty acids; SM, semimembranosus; SOD, superoxide dismutase; SPME, solid-phase microextraction; TAC, total antioxidant capacity; TBARSs, thiobarbituric acid reactive substances; TEs, Trolox equivalents; TEAC, Trolox equivalent antioxidant capacity; TI, thrombogenic index; TFC, total flavonoid content; TPAs, total phenolic acids; TPC, total phenol content; TTC, total tannin content; TVB-N, total volatile basic nitrogen; WBR, whey before ricotta; WPR, whey post ricotta; WYC, wine lees extract.
